# A Historical Twist on Long-Range Wireless: Building a 103 km Multi-Hop Network Replicating Claude Chappe’s Telegraph

**DOI:** 10.3390/s22197586

**Published:** 2022-10-06

**Authors:** Mina Rady, Jonathan Muñoz, Razanne Abu-Aisheh, Mališa Vučinić, José Astorga Tobar, Alfonso Cortes, Quentin Lampin, Dominique Barthel, Thomas Watteyne

**Affiliations:** 1 Orange Labs, 38240 Meylan, France; 2 Inria, 75012 Paris, France; 3 CITI, National Institute of Applied Sciences (INSA) Lyon, Inria, University of Lyon, 69621 Villeurbanne, France; 4 Wattson Elements/Falco, 75012 Paris, France; 5 Nokia Bell Labs, 91620 Nozay, France

**Keywords:** LPWANs, industrial Internet of Things, mesh networks, wide area networks

## Abstract

In 1794, French Engineer Claude Chappe coordinated the deployment of a network of dozens of optical semaphores. These formed “strings” that were hundreds of kilometers long, allowing for nationwide telegraphy. The Chappe telegraph inspired future developments of long-range telecommunications using electrical telegraphs and, later, digital telecommunication. Long-range wireless networks are used today for the Internet of Things (IoT), including industrial, agricultural, and urban applications. The long-range radio technology used today offers approximately 10 km of range. Long-range IoT solutions use “star” topology: all devices need to be within range of a gateway device. This limits the area covered by one such network to roughly a disk of a 10 km radius. In this article, we demonstrate a 103 km low-power wireless multi-hop network by combining long-range IoT radio technology with Claude Chappe’s vision. We placed 11 battery-powered devices at the former locations of the Chappe telegraph towers, hanging under helium balloons. We ran a proprietary protocol stack on these devices so they formed a 10-hop multi-hop network: devices forwarded the frames from the “previous” device in the chain. This is, to our knowledge, the longest low power multi-hop wireless network built to date, demonstrating the potential of combining long-range radio technology with multi-hop technology.

## 1. Introduction

Wireless connectivity has been increasingly deployed in diverse industrial and urban applications. It has enabled automated remotes and frequent monitoring of machines and smart meters. This increased adoption has challenged wireless communications to offer higher capacities and longer ranges at lower costs.

In Industry 4.0, wireless is a key component for enabling remote interaction with machinery. Sensors can report temperature, pressure, and vibration [1]. Early performance degradation signs can be detected and the machine can be maintained before anything breaks, with the promise of avoiding unplanned downtime. Predictive maintenance adds *prognosis* to *diagnosis* [2]. An industrial plant has hundreds of sensors or actuators connected to low power networks [3] deployed in a challenging environment. For example, an oil refinery can have more than one million devices spread over an area of several square kilometers, in an environment full of metallic structures [3]. This challenges the range of the wireless network. In these setups, wiring the sensors together is often not an option because of the installation complexity and operational hazards [3,4].

Long-range wireless communication is used to remotely read domestic utility meters (water, electricity, gas). The European Commission mandates their remote readings to save natural resources [5]. The monitoring infrastructure is necessarily wireless, as running a dedicated wired network between houses is too expensive [6]. Similar to industrial use cases, the distance between utility meters is a challenge for wireless, particularly as meters can be installed underground or behind concrete barriers.

These applications have the same challenge: “how can we extend the area these networks are deployed in without installing significant infrastructure?”. These networks typically use long-range radios in a star topology, or short-range mesh topologies. Both are limited by the fact that gateways need to be installed for the nodes to report data. Gateways are expensive to install as they need mains power and internet connectivity (Ethernet, cellular, etc.). The goal of both long-range radio technology and multi-hop mesh networking technology is to reduce the number of gateways installed.

Several radio technologies have been developed for low-power long-range wireless networking. The IEEE has standardized a set of 31 PHYs in the IEEE802.15.4g (2012) amendment [7]. They are based on three families of modulations: frequency shift keying (FSK), offset-quadrature phase shift keying (O-QPSK), and orthogonal frequency division multiplexing (OFDM). Long-Range (LoRa) is a proprietary PHY that has become pervasive in low-power long-range networks. It is based on chirp spread spectrum modulation and it is deployed in a star topology under the LoRa wide area network (LoRaWAN) protocol stack. Sigfox is a technology that was available recently for long-range low-power wireless networking. Similar to LoRa, it used a proprietary PHY and protocol stack and was based on star topologies. Cellular networks, standardized by the 3rd generation partnership project (3GPP), introduced narrow-band IoT (NB-IoT) for low-power wireless applications. It is provided as an integrated service in cellular base stations by “slicing” the network into machine-type communications and human-type communications. Recently, satellite communication solutions have been introduced for long-range low-power connectivity to help reach areas where no terrestrial coverage is available. These families of networks are limited by the installation costs of their infrastructure, whether gateways, cellular-based stations, or satellites.

This article recreates the Claude Chappe Telegraph by applying its concept to low-power wireless. Using 11 battery-powered IoT devices, we created a 103 km low-power wireless network. The contributions of this article are three-fold:

- We provide a historical overview of the development of the Chappe Telegraph.
- We describe how we translate that concept to today’s low-power wireless technology.
- We demonstrate a wireless network reaching 103 km, and discuss the challenges and the opportunities for long-range multi-hop mesh networks.

The demonstration presented in this article is the longest low-power wireless multi-hop network, to the best of our knowledge.

The remainder of this article is organized as follows. Section 2 presents a historical overview of the Chappe telegraph. Section 3 surveys current IoT technologies. Section 4 introduces the experimental setup we used to replicate the Chappe telegraph. Section 5 describes the experiment of building a 103 km low-power wireless network. Finally, Section 6 discusses the opportunities and challenges for long-range mesh networking.

## 2. Claude Chappe’s 1794 Telegraph

Telegraphy is a word from ancient Greek that means “remote writing”. Both the Roman and Persian empires had systems of remote signaling of simple information that date as far back as the second Century B.C. [8]. The intensity of conflicts in Europe that erupted at the end of the 18th century created an urgent need for faster communications. It was at that time that Claude Chappe, a French engineer, developed his telegraph.

Starting in 1792, France entered into conflicts on several fronts with Austria, Prussia, Russia, and Britain. As troops were deployed in different zones, fast relaying of news and commands was necessary for tactical reactions. Claude Chappe indicated: “it is imperative to establish a rapid communication network, with which we can orchestrate the movements, simultaneously, of a million men dispersed on an immense space as if they were at the same place” [translated] [9].

**Figure 1:**
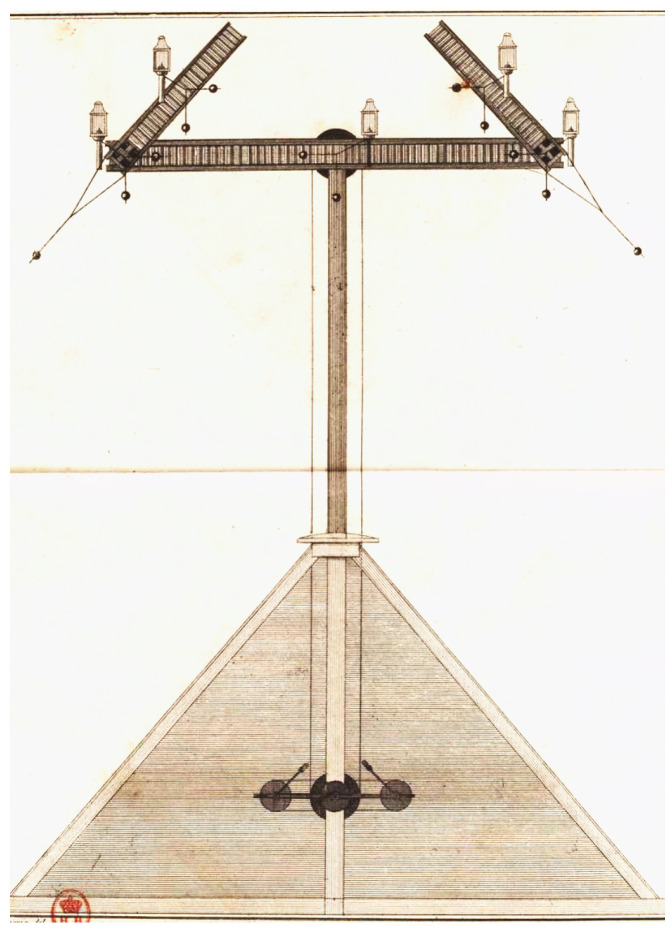
The Chappe telegraph adopted by the French state as depicted in Ignace Chappe’s book. Three arms were used to convey signals and each could rotate at steps of 45°. (Source: [9]).

In 1790, Claude Chappe created the first telegraph based on time-synchronized clocks to transmit information from one tower to the next. Afterward, he introduced a new design that did not require time synchronization and relied purely on optical observations. It relied on a wooden structure installed at the top of a tower, made up of one long beam and two shorter beams, see Figure 1. Operators inside each tower rotated the beams using pulleys, in steps of 45 degrees. This allowed for 4 positions of the long beam and 8 positions of each of the short beams, resulting in 256 symbols [8]. Information was encoded in pairs of symbols, resulting in a dictionary of 256×256 possible words. Messages were transmitted in a “multi-hop” manner: operators used binoculars to look at the position of the upstream tower and replicated it on theirs.

In September 1793, the General Assembly approved the establishment of a line of 15 towers from Paris to Lille. On 16 July 1794, the Paris-Lille line was completed and tested with 18 towers, covering a distance of 190 km with hops between 10 and 15 km. The transmission rate was estimated at one symbol per minute, i.e., one word every two minutes [8].

**Figure 2:**
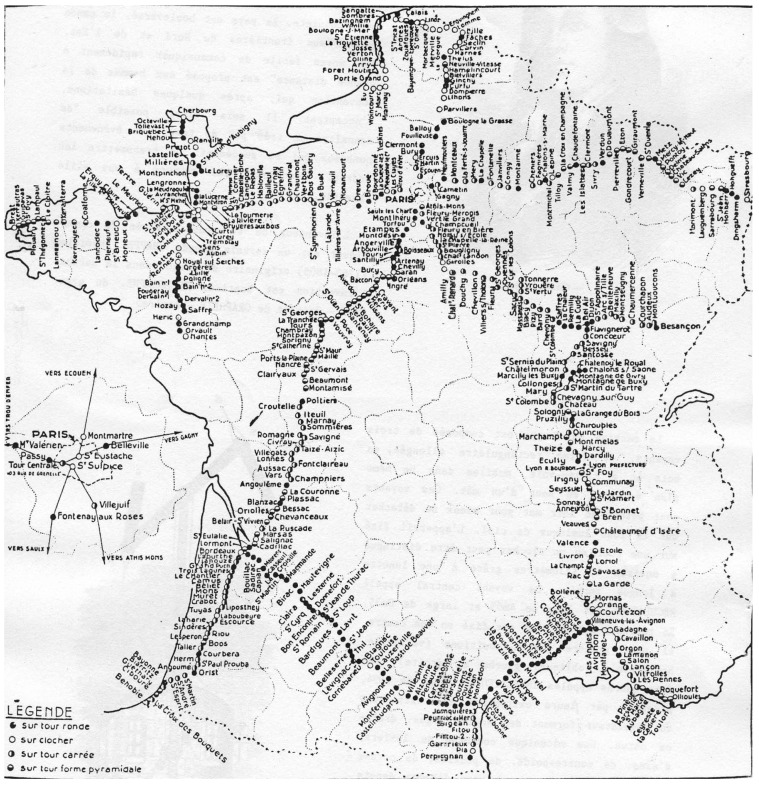
The Chappe Telegraph network deployed between 1794 and 1846. Each dot represents a tower. (Source: Cité des Télécoms).

Chappe introduced further improvements to his telegraph: the tower was redesigned to withstand higher winds, a portable version of the telegraph was introduced for in-battle communication, and lanterns were installed on the beams to allow for operation at night [9]. The deployment of the towers expanded rapidly under Napoleon Bonaparte as he realized their tactical advantage [8]. By 1846, the Chappe telegraph covered most of France (Figure 2).

The telegraph expanded to other countries [9]. It was experimented with in England, Turkey, Sweden, and Russia. It crossed the Mediterranean during the rule of Mohamed Aly Pasha, governor of Egypt, who wanted to establish a communication line between Cairo and Alexandria. He imported models from France and had his architect choose how to place the machines. The messages traveled between Alexandria and Cairo in 40 min, with a line of 17 stations covering nearly 180 km.

Samuel Morse experimented with using electricity to communicate, and, in 1837, he introduced a single-wire technique [8] that was far more economical than the Chappe telegraph. In 1849, the electrical telegraph was tested over large distances and fully patented, marking the beginning of the end for the optical telegraph. It was slowly adopted in Europe and France; by 1881, all optical telegraphs had been replaced by the electrical telegraph [8].

## 3. Survey of Current IoT Technologies

**Figure 3:**
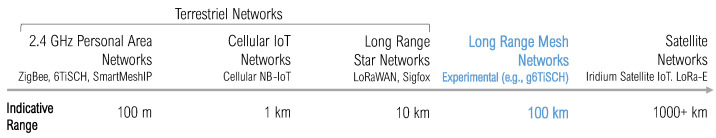
Existing low-power wireless technologies and their indicative ranges.

Different IoT technologies offer different trade-off points between the communication range, throughput, and power consumption. Figure 3 gives examples of these technologies, together with an indication of the communication range. This section provides an overview of single-hop networks and multi-hop networks. We discuss the contributions of this article in light of the state-of-the-art.

### 3.1. Single-Hop IoT Networks

This section discusses the four technologies of single-hop wireless networks for IoT: NB-IoT, cellular networks, LoRaWAN, Sigfox, and satellite networks.

Long-range connectivity is offered by cellular network operators using NB-IoT—an enhancement of long-term evolution (LTE). NB-IoT is standardized in Release 13 of the 3GPP [10]. It improves the communication range by combining sub-GHz frequencies (in the 900 MHz band) with a narrow channel bandwidth of 180 kHz [10]. It uses OFDM on top of O-QPSK modulation [11,12]. The communication range of NB-IoT is ≈1 km [13].

Another example is LoRaWAN, which uses a proprietary LoRa PHY [14]. LoRa uses chirp spread spectrum (CSS) FSK modulation, which makes it robust against multi-path fading, sub-GHz frequencies, and narrow-band channel bandwidths of 125 kHz (compared to 2 MHz for O-QPSK 2.4 GHz). Depending on regional regulations, the LoRa modulation may occupy channel bandwidths as narrow as 7.8 kHz. LoRa offers a range of bit rates, based on a spreading factor index (SF) of its CSS sweeps. The fastest bit rate of 5.4 kbps is achieved with SF-7 and the slowest bit rate of 293 bps using SF-12 (assuming 125 kHz bandwidth and 4/5 coding rate) [15]. Range tests show that LoRa PHY offers a 70% packet delivery ratio up to 10 km distance [16] in an urban setting without line-of-sight guarantees.

Sigfox was another LPWAN technology [17]. It featured sub-GHz operations, an ultra-narrowband channel bandwidth of 100 Hz, and a 100 bps bitrate [12]. Sigfox was an operated network: base stations were installed throughout the service area. However, due to the unstable market economy during the recent pandemic, Sigfox declared bankruptcy in early 2022 [18].

LPWAN technologies require base stations to provide connectivity to devices deployed around them in star topologies, typically within 10 km. Installing/maintaining such base stations is costly and may not be an option in remote areas or in complex critical infrastructures that do not allow invasive installations. Moreover, even as LPWANs show high link robustness outdoors or indoors [19,20], network coverage blind spots are inevitable (i.e., places where end devices are completely out of network range), such as in deep indoor devices (e.g., utility meters). An example of such blind spots of a LoRaWAN network was demonstrated in previous work [21]. In this case, it is often not economically viable to install a new base station only to service a few uncovered nodes. Users can still operate their own gateways for some technologies, such as LoRaWAN, but they need to provide their own network server and their own way of retrieving the received data. Therefore, only a minority of end users have the skills and resources to do that.

Low Earth orbiting (LEO) satellites orbit at an altitude between 160 and 2000 km, allowing them to offer network coverage to hundreds of square kilometers [22]. This allows them to be useful for remote sensing and monitoring systems spanning hundreds of kilometers. For example, they are suitable for IoT for over-the-horizon Maritime surveillance [23], environmental monitoring [24], emergency management, and smart grid monitoring [25]. LEO satellites follow two kinds of patterns: a Walker Star formation and a Walker Delta formation. In Walker Star, satellites orbit the earth in a 90° inclination, passing over both poles. They offer global coverage but fly over a given location infrequently, typically four times a day, with a total of 20 min per day of availability for a device. In Walker Delta, a satellite follows an inclined orbit close to the equator. This is interesting for populated areas around the equator but offers less global coverage. Direct connectivity to a satellite is available only once it is over the region of the device. There is a trade-off between network availability and constellation density (therefore, constellation and network cost).

Iridium is one technology for satellite IoT [26]. It relies on LEO satellites orbiting in a Walker Star formation [22] offering global coverage, with an orbital period of 100.13 min. The Iridium constellation contains 77 satellites. The technology relies on a combination of techniques for its PHY layers: an unmodulated tone, a unique word transmission in binary phase shift keying, and data payload transmission in O-QPSK. Reliability is achieved at the medium access control (MAC) layer by use of: (1) FDMA with 240 frequency channels and a 48 kHz channel bandwidth on the 1616 kHz baseband; and (2) TDMA with a 90 ms frame duration. Iridium devices can have as much as 10% of the lifetime of LoRaWAN or Sigfox devices when a message is sent every 10 min because, in part, of the long transmission procedure that can take between 6 and 20 s [26]. Therefore, their deployment is feasible only with solar power or with an electrical outlet. Similar satellite coverage solutions include the LoRaWAN-based Lacuna Space [27] and the proprietary Swarm Space network by SpaceX [28].

LoRa-E is an LPWAN technology designed for dense deployments and satellite connectivity [29]. It is an addition to the LoRa suite of modulations that uses long-range frequency-hopping spread spectrum (LR-FHSS) and provides a >155 dB link budget necessary for LEO satellites. In Europe, it uses a channel bandwidth of 137 or 336 KHz (compared to the typical 125 kHz for LoRa) and it divides the channel into sub-channels of 488 Hz of bandwidth (280 or 688 channels in the EU). Long-range and robustness improvements are achieved by a combination of transmitting duplicate headers, and fast hopping over a subset of the sub-channels, thereby allowing simultaneous transmissions in the same channel. Simulations show that LoRa-E increases the amount of network goodput by nearly two orders of magnitude compared to LoRa; therefore, it is a good candidate in dense environments [29].

Today, satellite connectivity is the only option when base stations cannot be installed.

### 3.2. Multi-Hop IoT Networks

In this section, we provide an overview of the two kinds of multi-hop networks: short-range and long-range. Some IoT personal area networks (PANs) operate at 2.4 GHz. One example is the IEEE O-QPSK 2.4 GHz PHY of the IEEE 802.15.4 standard. It uses a 2 MHz channel bandwidth and is the PHY of different protocol stacks [30]. The communication range of this PHY is in the order of tens of meters depending on the deployment environment. Networks using this PHY typically use multi-hop mesh topologies to extend their coverage. This includes Internet engineering task force (IETF) 6TiSCH standard protocol stack (IPv6 over the TSCH mode of IEEE 802.15.4e) [31], ZigBee [32], and SmartMesh IP [33].

Smart meter regulations in Europe mandate member states to provide smart meter capabilities as alternatives to old meters “unless […] this is not cost-effective in relation to the estimated potential savings in the long term” [5]. Since cost-effectiveness is key to IoT networks, high-maintenance gateways and satellites may not be cost-effective, even if they are energy efficient [34]. Some applications have strict constraints on message frequency. For instance, peach orchards require regular reporting of humidity to predict frost, otherwise peach farmers can lose all of their crops [35]. This was witnessed as Argentina lost as low as 85% of its peach crops in 2013. Therefore, when such use cases are out of coverage of 10-km scale terrestrial LPWANs, it would be unpractical to use satellite devices, which offer only an average of 20-min of availability every day [26].

Long-range low-power wireless mesh networking can be an option in such cases. They allow a flexible coverage extension using low-cost battery-powered devices. The IEEE already adopted a family of 31 physical layers for long/short-range IoT connectivity [7]. Their performances were evaluated in an exhaustive range of test campaigns in indoor, urban, agricultural, and remote area scenarios [36]. They offered the bitrate as fast as 800 kbps using OFDM 868 MHz and a range as far as ≈14 km when using FSK 868 MHz.

Recent research has provided experimental evaluations of both single-hop and multi-hop IoT networks. Cattani et al. [37] showed the performances of single-hop LoRa links in three scenarios: indoors, outdoors, and underground. They ran the setup on a university campus and they showed the link quality in the received signal strength indicator (RSSI) and packet reception ratio (PRR). They reported the performance in ranges up to 135 m. They showed that the fastest bitrate PHY had a mean PRR that was only 10% less than the slowest bitrate PHY. Hardie and Donald [20] ran a performance evaluation campaign of single-hop LoRa links in the 433 MHz band in an underground communication scenario and they reported the link quality in RSSI and signal-to-noise ratio (SNR). They showed a maximum range of the LoRa link of 200 m in an underground to above-ground link. Similarly, Cecilio et al. [38] ran experimental evaluations of single-hop LoRa links for flood detection scenarios by placing the transmitters and the gateway at the sides of a lake. They reported link qualities in terms of RSSI, PRR, and SNR for three settings: low tide, high tide, as well as in rural settings. They showed a maximum range of the LoRa link up to 1300 m during the high tide when the transmitter was 2 m above ground but this range was reduced to 650 m when the transmitter was 1 m or less above ground. Valecce et al. [19] tested NB-IoT connectivity in an agricultural setup. They showed that NB-IoT maintained connectivity at 83% PRR to an underground cellar and up to 90% PRR in an open terrace. However, they did not report the maximum range to the base station since this was not provided by that NB-IoT setup. Liando et al. [39] performed the longest single-hop tests of LoRa links, to our knowledge. The authors ran LoRa links in three scenarios: outdoor line-of-sight, outdoor non-line-of-sight, and indoors. They showed (experimentally) that packet reception was possible at a maximum of 10 km using LoRa SF-7. Using regression analysis, they estimated that LoRa could reach up to 17 km using SF-12 (in the outdoor line-of-sight scenario). Finally, Parri et al. [40] carried out performance evaluations of LoRa links at 8.3 km of distance in a marine environment. The authors found that using LoRa SF-7 offered the best trade-off between link robustness and power consumption compared to higher SFs, an observation confirmed also in [37].

Further research provided experimental evaluations for multi-hop IoT networks. Tran et al. proposed a protocol stack for a multi-hop LoRa network based on time-slotted MAC and demonstrated the reliability of 95% in a two-hop network [41]. The authors evaluated it in a simulation in an area of 800×800m${}^{2}$ and experimentally in a 400×400m${}^{2}$ area, using SF-7 LoRa PHY. Mai et al. proposed a time-slotted MAC for the multi-hop LoRa network with a focus on minimizing latency using parallel transmissions [42]. The authors ran an experimental evaluation using the SF-7 LoRa PHY in a 400×400m${}^{2}$ area. Similarly, Basili et al. [43], provided an architecture for a multi-hop linear LoRa network that acted as an extension for a LoRaWAN gateway. They ran the experimental setup and they demonstrated the feasibility of the system in a four-hop network. In previous research, we used the IETF standard 6TiSCH protocol stack with the long-range FSK 868 MHz PHY [44]. We then proposed a generalized 6TiSCH protocol stack (g6TiSCH) to integrate any combination of long-range and short-range PHYs in the same network [45]. The g6TiSCH architecture was evaluated on an indoor testbed of 36 motes in a 100 m${}^{2}$ area office setup. We showed how a network could improve its reliability by combining long-range and short-range radios in a mesh topology, using heterogeneous 6TiSCH slotframes [46].

The power consumption of the technologies discussed in this section (Figure 3) increases depending on several factors, such as: the current draw of the used radio chips, transmit power, MAC layer configuration, and link quality. Generally, it ranges from tens of milliamps for a 2.4 GHz mesh network or a LoRa transceiver [15,33,44], to hundreds of milliamps for satellite IoT transceiver [47].

The articles cited in this section demonstrate the growing interest in combining long-range PHYs with multi-hop connectivity. There are two common limitations to these articles. First, they did not test the maximum line-of-sight connectivity between the nodes as the maximum range achieved experimentally was for LoRa PHY at 10km [39]. Second, the outdoor long-range setup experiments with proprietary PHYs (i.e., LoRa or NB-IoT), did not consider the potential benefits of standard IEEE 802.15.4 PHYs. In this article, we went one step further by building a 103-km low-power wireless multi-hop network to demonstrate the potential of long-range low-power wireless full-fledged mesh networking. We used the standard IEEE 802.15.4 FSK 868 MHz PHY and we demonstrated that the range limit in line-of-sight could reach up to 12.7 km in the single-hop distance.

## 4. Experimental Setup

This section presents the planning of the experimental setup in two parts: configuring the network specifications (Section 4.1), and planning the geographical deployment (Section 4.2). A major challenge was to ensure reliability and speedy installation. The full demonstration had to be done in a single day. We only had 6 h of daylight and needed to deploy 11 balloons along a 103 km route. Reliability and speed were our main objectives.

### 4.1. Network Configuration

**Figure 4:**
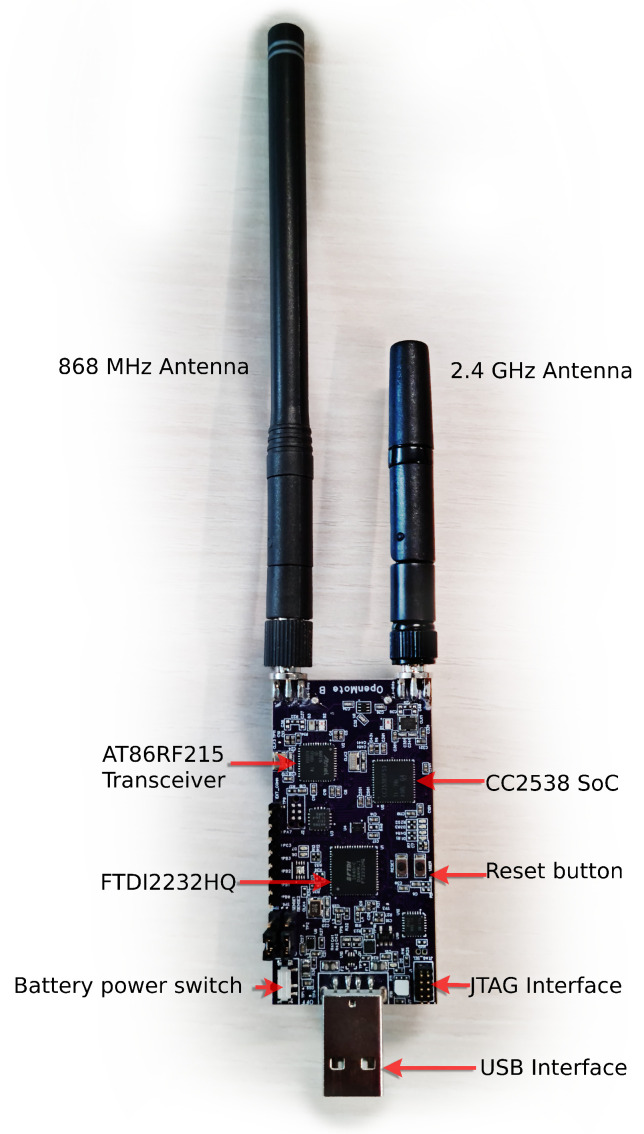
The OpenMote Bused in parts of the experiment.

We used two sets of hardware, the OpenMote B Figure 4 and the proprietary Falco hardware. We use the generic term “mote” to refer to either. The OpenMote B [48,49] features the Texas Instruments CC2538 System-on-Chip [50] and the Atmel AT86RF215 radio chip [51]. The latter implemented all IEEE 802.15.4g PHYs; we used the IEEE standard FSK Option 1 modulation with forward error correction at the 868 MHz band at 50 kbps. This PHY layer was tested in previous research and showed the highest robustness in the family of the IEEE 802.15.4g PHYs [36].

**Figure 5:**
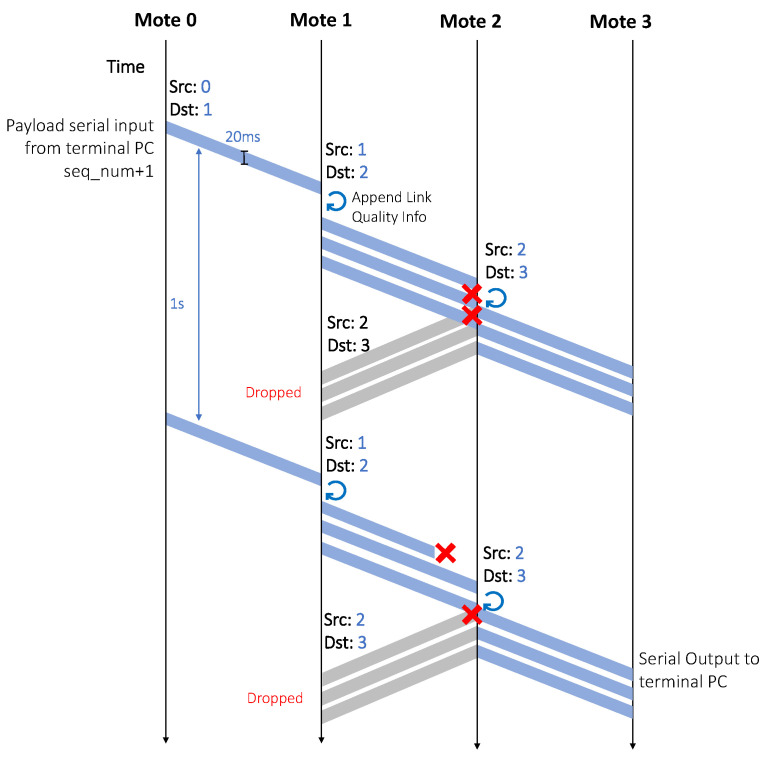
The communication protocol relays transmitted each received packet three times to increase reliability.

We used a specially-crafted multi-hop communication protocol, shown in Figure 5. The root node was set to re-transmit a frame every second. At the MAC layer, each relay listened for packets from the previous hop. The relay transmitted the received packet three times, every 20 ms.

A packet included the source and destination fields. Each device statically allocated a unique identifier, from 0 to 10. Device 0 was the root. Each relay incremented the source and destination fields in the relayed packet, resulting in multi-hop routing. This was used to prevent loops and backward-relaying because a node dropped any packet that was not destined for it. For debugging, relay nodes appended the RSSI of the received packet to the relayed packet.

**Figure 6:**
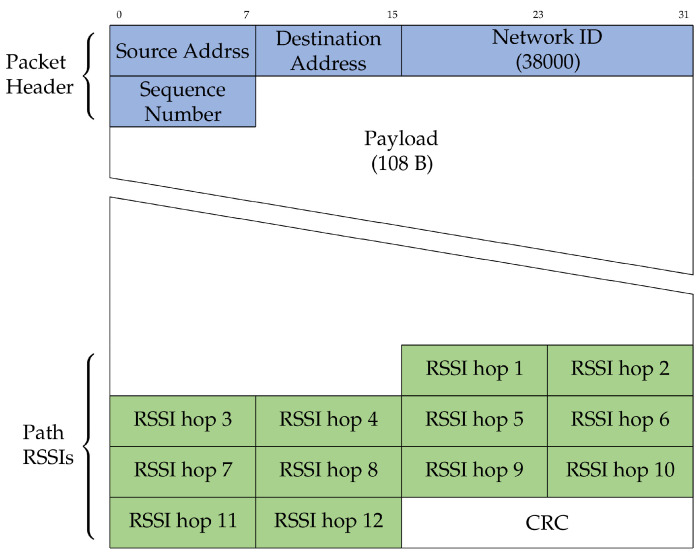
Format of the packet format used. Source and destination addresses are used for hop-by-hop routing.

The packet format (Figure 6) consisted of three main parts: the packet header, payload, and footer containing the captured RSSIs along the path. The header contained the source and destination fields, a network ID used to filter out any packets received from outside the network, and a sequence number used to filter out duplicate packets. At the footer, 12 B were used to store RSSI packets of each of the 12 hops (10 hops between balloons and two extra hops to the terminal computers on the ground at both ends) in the network, as well as a 2 B CRC check. A packet had a maximum of 127 B.

### 4.2. Network Planning

**Figure 7:**
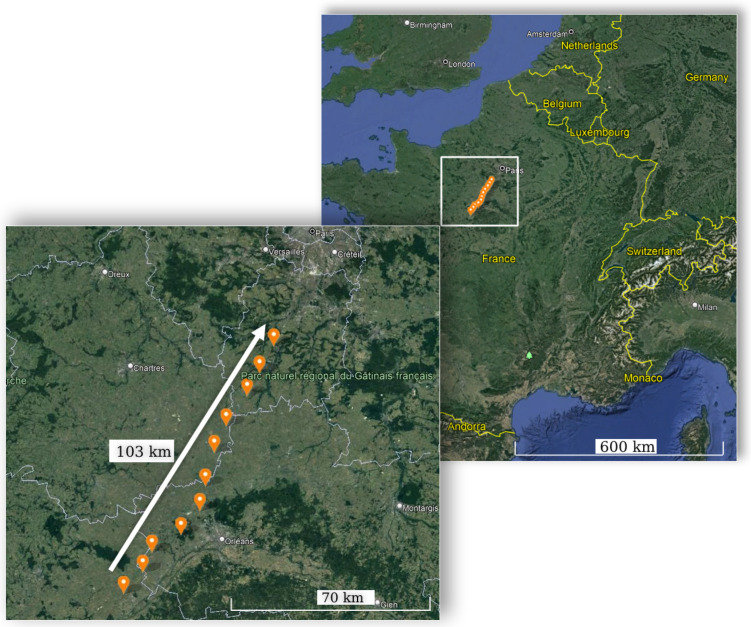
Location of the experiment in the southwest of Paris.

We selected a set of locations in the southwest of Paris from the historical map of Claude Chappe towers (Figure 2). The locations are shown in Figure 7, corresponding to the following villages (from north to south): Torfou, Etampes, Angerville, Arbouville, Toury, Artenay, Chevilly, Bucy, Baccon, Cravant, Séris.

**Figure 8:**
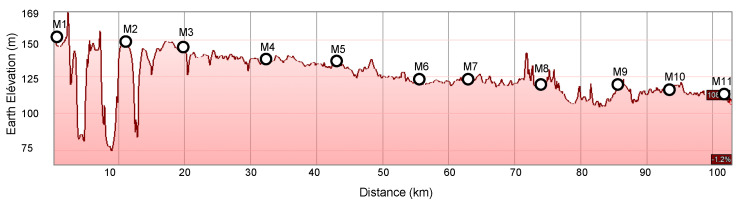
Terrain elevation is an important factor when selecting locations.

Two factors affected the selection of the exact coordinates for positioning the motes. The first challenge was the variation in the terrain altitude. Figure 8 shows the terrain altitude of the covered distance and the selected locations of the motes. For example, at the first hop, the altitude varied between 75 and 170 m at a distance of 10.5 km. This was enough of a difference to have it impact the communication range; the terrain profile is an important factor to take into account when selecting locations.

The second challenge was to ensure the distances between the motes were within the communication range of the motes. We needed to find locations close enough to one another while avoiding densely populated areas (to avoid inconveniencing residents). Table 1 outlines the distance of each hop along the path, resulting in a total of a 103 km network using 11 motes.

**Figure 9:**
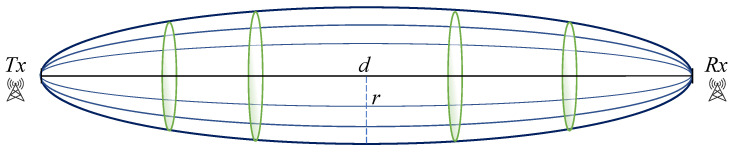
Illustration of the Fresnel zone between the transmitter and receiver.

After selecting the locations, we needed to place the motes at appropriate heights that allowed for line-of-sight visibility. We were particularly aware of the Fresnel zone clearance: the line-of-sight between the transmitter and receiver needed to be clear of any obstructions by a certain distance called the Fresnel radius. The longer the range, the larger the radius that needed to be clear of obstructions [52]. The Fresnel zone is an elliptical-shaped zone between the transmitter and the receiver, as seen in Figure 9. Similar to the research in [38,40], we calculated the required Fresnel radius clearance. For a distance of *d* km between the transmitter and the receiver, and using frequency *f* GHz for communication, the maximum radius of the ellipse *r* was at the center of the ellipse and it could be calculated as in Equation (1). Using 868 MHz frequency and the distances of each hop, the minimum required Fresnel clearance is shown in Table 1 for each hop. Therefore, a theoretical minimum of 33 m of clearance above ground was needed. Further clearance was also required to account for intermediate bumps in terrain elevation that could reach up to 15 m as seen in the hop between mote 7 and mote 8 in Figure 8.

(1) $$r=8.656\sqrt{d/f}$$

With distances between motes up to 13 km (Table 1), and as we used the 868 MHz frequency band, a clearance of 33 m above ground or obstructions was needed, according to the Fresnel zone formula [52].

## 5. A 103-km Wireless Network

**Figure 10:**
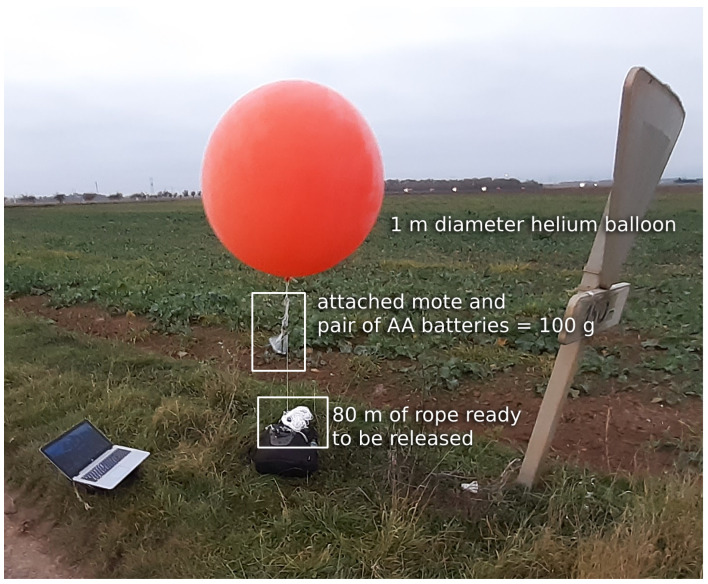
An OpenMote Bwas attached to a helium balloon.

To ensure a good communication range, we used helium balloons to lift the motes in the air. These needed to be large enough to keep the mote high enough even in the event of wind. We tested latex balloons with a diameter between 80 and 100 cm, and chloroprene balloons with a 120 cm diameter. We conducted the experiment in clear weather, at 14 °C, with wind speeds between 12 and 15 km/h, and wind gusts up to 18 km/h. We used helium with 97% purity. The payload consisted of a mote (either OpenMote B or Falco) powered by a pair of AA batteries, weighing a total of ≈100 g. We selected the chloroprene balloon, which we inflated to 100 cm diameter; even with an 80 m rope altitude, they stayed at 50 m or higher in the wind conditions of the day. Figure 10 shows one of the balloons used.

**Figure 11:**
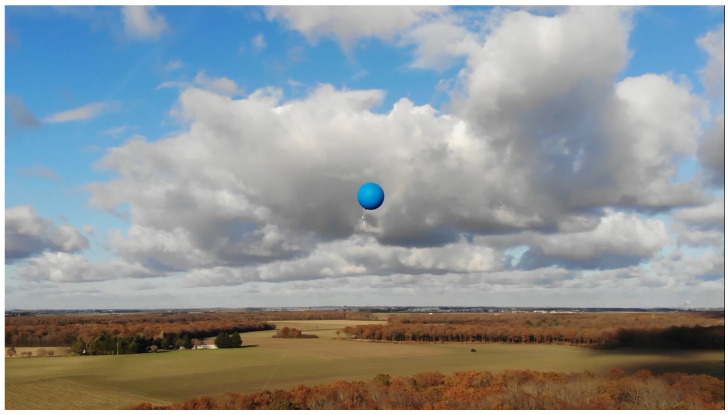
One out of 11 balloons carrying a mote.

At each location, we inflated the balloon, attached the mote under it, and released up to 80 m of the rope as shown in Figure 11. This allowed each balloon to be within the line of sight of the next, higher than the intermediate trees or the occasional house while respecting the Fresnel zone clearance. The experiment was conducted in rural areas where no trees or buildings were more than 20 m high. Having the balloon float at 50 m left sufficient space for the Fresnel zone at each hop. Therefore, we released enough rope so the balloon would float at 50 m or higher. Depending on the location, we attached the rope to a tree branch, a sign, or anything sturdy.

We used two cars for the experiment, driving about 10 min from one another. The first car was in charge of inflating the balloon at the location, the second of attaching and testing the mote. A person stayed at the initial location (Torfou) with a mote connected to a laptop; he could type in a message that would be sent over the chain of motes. At each new location, we used another laptop attached to a mote to verify the message reached that far. We had no technical difficulty throughout the experiment; communication continuously worked at each location. We also had no balloon- or battery-related failures. The deployment of all 11 balloons took 6 h.

**Figure 12:**
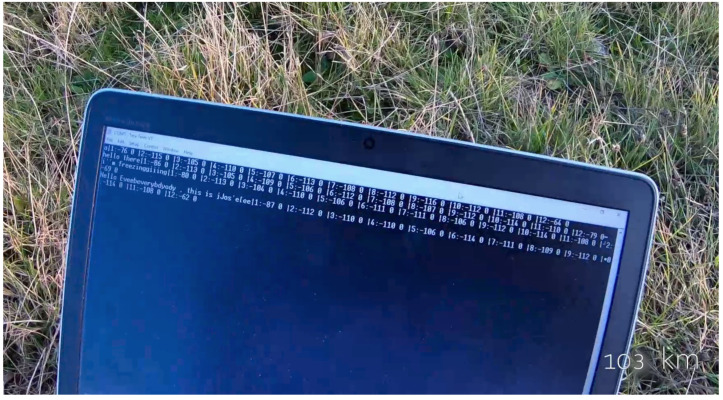
Captured packets 103 km away from the transmitting computer.

Figure 12 is a picture of the laptop screen at the last location (the village of Séris), 103 km away from the transmitting computer. It shows the messages sent by the person in Torfou, as well as the RSSI at each of the hops.

**Figure 13:**
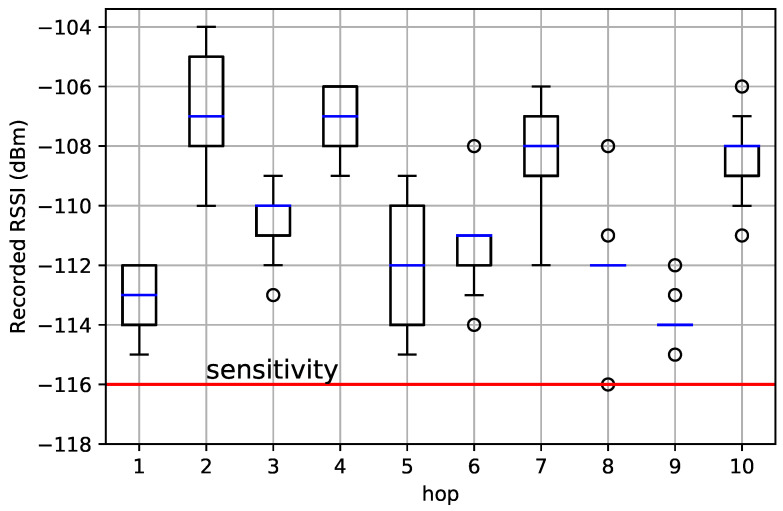
The RSSI at the receiving mote of each hop, when using the OpenMote B. The red bar shows the sensitivity of that radio in the configuration we used: we needed the RSSI of each hop to be above that.

Figure 13 shows the recorded RSSI at each hop when using the OpenMote B. The sensitivity of the AT86RF215 when using the FSK PHY is −116 dBm; it is represented as the red bar in Figure 13. We needed the RSSI at each hop to be above that sensitivity. Figure 13 shows the RSSIs of 90 packets captured when we completed the 6 hours of deployment as box plots (median in the red bar and the box limits representing 25% and 75% of the data). We can see that hops 1, 5, and 8 were the “weakest” (the RSSI closest to sensitivity) while hops 2, 7, and 10 were the “strongest” (the motes could have been separated more).

The captured RSSIs showed promising link budgets of the standard FSK 868 MHz link at low-power consumption. The longest hop in the network was hop 3 with a 12.7 km distance. The packets captured at that hop showed median RSSI that was 6 dBm above the receiver sensitivity. It is worth noting the low-power consumption characteristics of the AT86RF215 chip used in this experiment: with 2.5V supply voltage, the current draw was 67 mA in transmission and 28 mA in receive mode [51]. When a similar test was conducted using LoRa SF-7 in line-of-sight, no packets were delivered beyond the 10 km range using the SX1276 radio chip [39]. Even though FSK 868 MHz is nearly five times higher in the data rate than LoRa SF-7, it still maintained packet reception up to 12.7 km with 6 dBm above the receiver threshold. This can be attributed to differences in experimental setups or transmit power configurations. This encourages further experimental evaluations, specifically of FSK 868 MHz compared to LoRa SF-7, especially as LoRa SF-7 is observed to offer the best trade-off between power consumption and link robustness in LoRa configurations [37,40].

## 6. Conclusions

Wireless networking has become an essential technology that serves several critical activities, such as smart industry, precision agriculture, and utility metering. In this article, we presented a historical overview of the development of long-range telecommunications by Claude Chappe. We discussed how long-range telecommunications through the Chappe telegraph paved the way for modern telecommunications.

As an homage to the Chappe telegraph, we replicated its vision, replacing its visual component with long-range radio communication. We demonstrated a 103 km network that we deployed in the southwest of Paris on a portion of a historical Chappe telegraph line.

The article does not argue for the specific PHY layer we used, nor proposes a specific technical architecture, as we focused on the simplicity and reliability of the network for the sake of the experiment. It serves as a strong highlight of the incredible potential of pushing the boundaries of long-range IoT technologies. Today, solutions such as LoRaWAN are, in fact, incredibly simple: motes transmit their data directly to a nearby base station. These technical choices are rather underwhelming, as they do not take advantage of many innovations in multi-hop mesh networking.

Moreover, while the protocol implemented is simplistic, it shows how a 10 km IoT technology can be used in a 100 km deployment. This is, we believe, where the road lies ahead.

Imagine you are a network operator in charge of the wireless connectivity that is meant to automatically read electricity meters. While the long-range IoT network you installed works well for most of your meters, approximately 1% of them are installed in “deep indoor” environments, such as in underground parking garages. Moreover, in your medium-sized city, this is over 1000 m scattered around. You could of course install many more gateways. Besides the obvious costs of their installation, it is the lengthy trial-and-error, the repeated repositioning, and the unhappy customers that are the real costs of this approach. The more sensible approach, we argue, is to augment the long-range technology with multi-hop capabilities: have motes “help out” the ones that are out of range of the gateway by relaying their data.

While we do not intend to provide a detailed performance evaluation of the network, with an exhaustive range-testing campaign, we find these initial results encouraging to explore the full potential of the standard IEEE 802.15.4g PHYs as opposed to proprietary technologies. Since we had successful packet receptions at distances up to 13 km with 6 dBm above the receiver threshold, we wonder if a standard PHY, such as FSK 868 MHz, can offer a reasonable trade-off between range and chip costs, due to the fact that it is a non-proprietary PHY. This may provide network architects with more diverse options for their networks with IEEE standard PHYs in addition to the proprietary LPWAN technologies.

## Figures and Tables

**Table 1:** Length of each hop in the network.

Hop		Distance	Fresnel Clearance
1	Torfou–Etampes	10.48 km	30.1 m
2	Etampes–Angerville	8.97 km	27.9 m
3	Angerville–Arbouville	12.67 km	33.1 m
4	Arbouville–Toury	10.25 km	29.7 m
5	Toury–Artenay	11.86 km	32.0 m
6	Artenay–Chevilly	8.74 km	27.5 m
7	Chevilly–Bucy	10.69 km	30.4 m
8	Bucy–Baccon	11.57 km	31.6 m
9	Baccon–Cravant	7.93 km	26.2 m
10	Cravant–Séris	9.97 km	29.3 m
**total distance**		**103.13 km**	

## Data Availability

Not applicable.
